# Hydraulic forces contribute to left ventricular diastolic filling

**DOI:** 10.1038/srep43505

**Published:** 2017-03-03

**Authors:** Elira Maksuti, Marcus Carlsson, Håkan Arheden, Sándor J. Kovács, Michael Broomé, Martin Ugander

**Affiliations:** 1 Department of Clinical Physiology, Karolinska Institutet, and Karolinska University Hospital, Stockholm, Sweden; 2 Department of Medical Engineering, School of Technology and Health, KTH Royal Institute of Technology, Stockholm, Sweden; 3 Lund University, Skane University Hospital, Department of Clinical Sciences Lund, Clinical Physiology, Lund, Sweden; 4 Department of Internal Medicine, Cardiovascular Division, Washington University School of Medicine, St. Louis, Missouri, USA; 5 Anaesthesiology and Intensive Care, Department of Physiology and Pharmacology, Karolinska Institutet, Stockholm, Sweden; 6 ECMO Department, Karolinska University Hospital, Stockholm, Sweden

## Abstract

Myocardial active relaxation and restoring forces are known determinants of left ventricular (LV) diastolic function. We hypothesize the existence of an additional mechanism involved in LV filling, namely, a hydraulic force contributing to the longitudinal motion of the atrioventricular (AV) plane. A prerequisite for the presence of a net hydraulic force during diastole is that the atrial short-axis area (ASA) is smaller than the ventricular short-axis area (VSA). We aimed (a) to illustrate this mechanism in an analogous physical model, (b) to measure the ASA and VSA throughout the cardiac cycle in healthy volunteers using cardiovascular magnetic resonance imaging, and (c) to calculate the magnitude of the hydraulic force. The physical model illustrated that the anatomical difference between ASA and VSA provides the basis for generating a hydraulic force during diastole. In volunteers, VSA was greater than ASA during 75–100% of diastole. The hydraulic force was estimated to be 10–60% of the peak driving force of LV filling (1–3 N vs 5–10 N). Hydraulic forces are a consequence of left heart anatomy and aid LV diastolic filling. These findings suggest that the relationship between ASA and VSA, and the associated hydraulic force, should be considered when characterizing diastolic function and dysfunction.

The mechanisms involved in left ventricular (LV) diastolic filling and their relative importance remain an area of active investigation^1^,^2^,^3^,^4^,^5^,^6^,^7^,^8^. Understanding the physiology of normal LV filling can advance characterization of the pathophysiology of diastolic dysfunction and consequently help in identifying optimal diagnostic metrics as well as targets for treatment. The main mechanisms known to act during LV diastolic filling are active relaxation^4^, restoring forces generated by elastic energy stored within the myocardium^1^,^9^,^10^ and atrial contraction.

At the end of LV systole, calcium ions are pumped back into the sarcoplasmatic reticulum, allowing for actin and myosin filament cross-bridges to uncouple and slide back to their initial position. This process is referred to as “active relaxation” since it requires energy in the form of adenosine triphosphate. Active relaxation is not instantaneous, and thus takes time to occur. Consequently, residual contractile forces are present early on during isovolumic relaxation upon the onset of diastole before all active relaxation has had a chance to occur, and this incomplete relaxation initially impedes LV myocardial fibers lengthening^2^.

Simultaneously with active relaxation, restoring forces act during LV diastole. These forces are generated by the myocardium and are attributed to the giant intracellular protein titin^9^, the extracellular matrix including collagen and elastin^11^, and the visceral pericardium^12^. On the molecular scale, titin is a bidirectional spring. When this molecular spring is displaced from its equilibrium position, either by compression or stretching, it generates an opposing force that brings it back toward its equilibrium length. This is the force responsible for the sliding of uncoupled actin and myosin filaments back to their equilibrium position and has been referred to in the literature with different terminologies including *restoring force*^9^,^13^, *elastic recoil*^14^,^15^ and *passive tension*^10^. For clarity, we classify and refer to these occurrences as follows: (i) during contraction the myocardium stiffens and compresses its internal elements, thereby storing elastic potential energy (*restoring force*); (ii) during myocardial relaxation this potential energy is converted to kinetic energy, by movement of the myocardium back to its resting length (*elastic recoil*); (iii) when the myocardium is elongated beyond its resting length, a force is generated to bring it back to equilibrium or oppose further lengthening (*passive tension*). The passive tension is also related to what is referred to as *passive myocardial stiffness*. Granzier and Irvin^10^ experimentally showed in rat cardiac muscle that there are three main contributors to passive myocardial stiffness: intracellular titin, extracellular collagen and intermediate filaments. Titin plays a more important role at short sarcomere working lengths, collagen at longer sarcomere working lengths, and the intermediate filament accounts for about 10% of the passive stiffness. Furthermore, the influence of the stiff collagen increases in pathological conditions^16^ and ageing^3^,^17^. The pericardium and the heart’s surrounding tissues, including the lungs and the diaphragm, might also accumulate a smaller amount of elastic energy during LV systole which is returned during diastole^18^.

To date, there is no method that can quantify and differentiate between active myocardial relaxation and the different elastic forces^19^, since all mechanisms manifest simultaneously during isovolumic relaxation and subsequent LV filling. Both experimental^2^ and theoretical studies, based on physical and mathematical modelling^13^,^20^ have sought to elucidate the mechanisms governing isovolumic relaxation^21^ and filling^13^,^20^.

We hypothesize the existence of an additional mechanism involved in LV filling, namely, a hydraulic force contributing to the atrioventricular (AV) plane motion in the apex-to-base direction. Hydraulic forces are based on Pascal’s principle, and have previously been hypothesized to contribute to diastolic filling^22^,^23^,^24^. The hypothesis has been tested using a mathematical model, where the AV plane was treated as a piston unit with two different areas^25^. In that model, the hydraulic force was assumed to be the only contribution to diastolic filling. Despite this simplification, simulations produced pressure and flow changes throughout the cardiac cycle within physiological ranges, suggesting that this force must be of a similar magnitude to other forces involved. In order to generate a net hydraulic force in the apex-to-base direction, a difference in short-axis area between the LA and the LV is required. However, *in vivo* measurement and comparison of the LA and LV short-axis areas have not been reported.

Therefore, we hypothesize that if the atrial short-axis area (ASA) is smaller than the ventricular short-axis area (VSA), it thus provides the anatomical basis for the presence of a hydraulic force during diastolic filling. The aims of this study were to (a) illustrate the effects of hydraulic forces in an analogous physical model of the LA and LV, (b) measure and compare the ASA and VSA in healthy volunteers, and (c) calculate the magnitude of the hydraulic force.

## Results

### Physical Model of the Hydraulic Forces aiding Left Ventricular Filling

**Figure 1:**
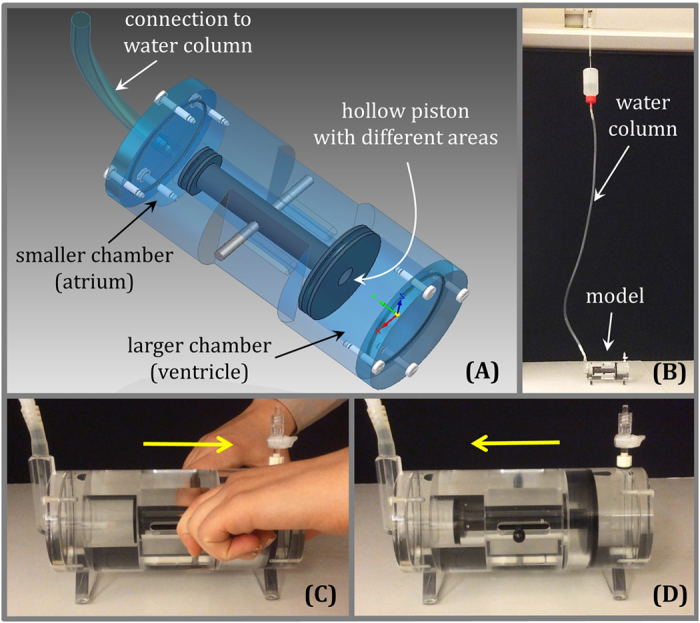
The physical model with a moving hollow piston illustrating hydraulic forces analogous to those present in the left atrium and left ventricle of the heart. (**A**) Three-dimensional drawing of a model constructed such that the larger chamber representing the ventricle is connected to the smaller chamber representing the atrium, through a hollow piston representing the mitral valve. The piston has two different areas at its extremities, representing the atrial short-axis area (ASA, the smaller area) and the ventricular short-axis area (VSA, the larger area). The water in the two chambers is in free communication via the conduit along the longitudinal axis of the hollow piston. Consequently, the pressure in the two chambers is the same and the liquid can move freely from one chamber to the other. The difference in area between the two extremities of the piston creates the basis for the generation of a hydraulic force. The longitudinal direction represents the apex-to-base direction and the open conduit represents the open mitral valve. (**B**) The model is pressurized by a water column with a height of approximately 140 cm connected to the smaller chamber. (**C**) The piston is manually moved towards the largest chamber. This phase is analogous to ventricular contraction when the atrioventricular plane moves towards the apex. (**D**) The piston is then released and freely moves back towards the smallest chamber under the action of hydraulic forces. This phase represents ventricular filling, when the atrioventricular plane moves towards the base, which in this model is caused by hydraulic forces alone. Please see the video of the moving model that is available as an online supplemental file accompanying this article.

For a more comprehensive description of the theoretical background governing the main findings presented below, please see *Methods - Theoretical Background Related to Hydraulic Forces*. The physical model illustrates the effect of hydraulic forces displacing a hollow piston (Fig. 1 and Supplemental Video S1). The model is pressurized through a hose by a water column and the same pressure acts in the two chambers since the piston is hollow. An external force analogous to ventricular contraction can be applied to pull the piston toward the chamber with the larger cross-sectional area (corresponding to the ventricle). During this contraction, a volume of water in the larger chamber is displaced through the hollow piston, into the smaller chamber and into the water column. Once released, the larger chamber fills through the movement of water through the hollow piston, and the piston spontaneously moves towards the chamber with the smaller cross-sectional area (corresponding to the atrium), mimicking the return of the AV plane in the apex-to-base direction during diastole, and solely driven by hydraulic forces.

### *In Vivo* Measurements

**Figure 2:**
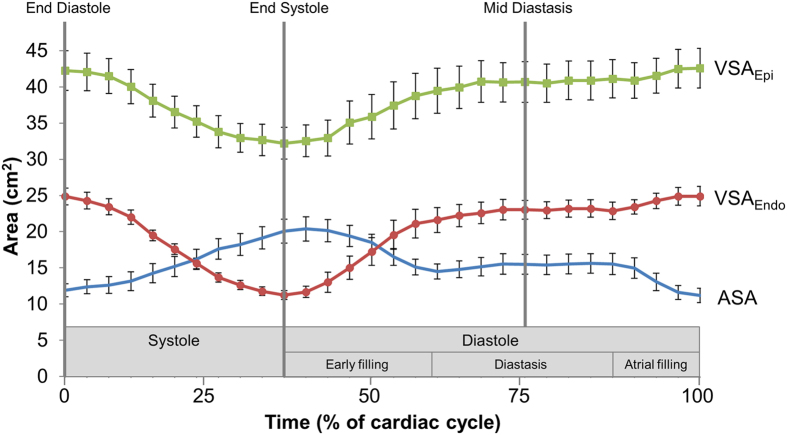
Short-axis areas of the left atrium and left ventricle over time. The three curves represent the mean for all subjects (n = 10) of the atrial short-axis area (ASA), the ventricular endocardial short-axis area (VSA_Endo_) and the ventricular epicardial short-axis area (VSA_Epi_). Vertical lines denote end diastole, end systole, and mid diastasis. Note that the VSA_Epi_ is larger than the ASA for the entire duration of diastole, and that the VSA_Endo_ is larger than the ASA for the majority of the duration of diastole. The error bars denote the standard error of the mean.

The changes of ASA, VSA_Endo_ and VSA_Epi_ during the cardiac cycle for all subjects are shown in Fig. 2. The VSA_Epi_ is larger than the ASA during all of diastole. The VSA_Endo_ is larger than the ASA for 75% of the diastolic phase, i.e. from 53% to 100% of the cardiac cycle in Fig. 2.

**Figure 3:**
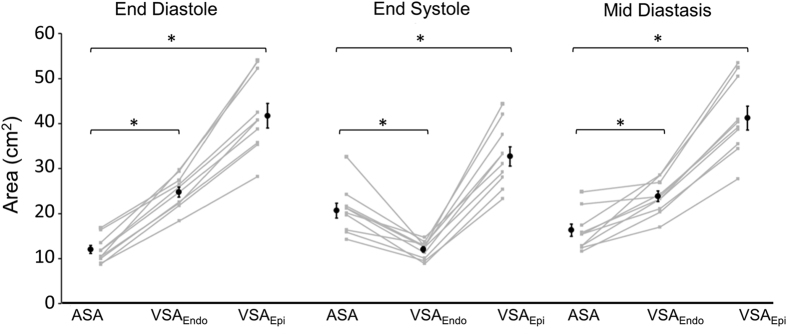
Comparison between the atrial short-axis area (ASA), the endocardial and epicardial ventricular short-axis area (VSA_Endo_ and VSA_Epi_) for each subject at three time points during the cardiac cycle (end diastole, end systole, and mid diastasis). The back dots represent the mean for all subjects and the error bars denote the standard error of the mean. *Denotes p = 0.02.

The relationship between ASA, VSA_Endo_ and VSA_Epi_ for each subject at three different time points (end diastole, end systole and mid diastasis) is shown in Fig. 3. At end diastole and mid diastasis, both VSAs are larger than ASA (p = 0.02). At end systole, VSA_Endo_ is smaller than ASA (p = 0.02) whereas VSA_Epi_ is larger than ASA (p = 0.02).

The difference between VSA_Endo_ and ASA at mid diastasis was 7.7 ± 1.4 cm^2^ yielding a hydraulic force of 1.0 ± 0.2 N. The difference between VSA_Epi_ and ASA at mid diastasis was 25.4 ± 2.3 cm^2^ yielding a hydraulic force of 3.4 ± 0.3 N. By comparison, the peak driving force of LV filling was estimated to be between 5–10 N (see also Methods, *Comparison between Peak Driving Force and Hydraulic Force*), and thus the hydraulic force (1–3 N) represents between 10–60% of the peak driving force.

## Discussion

The main finding of this study is that the ASA is smaller than the VSA for the vast majority of the duration of diastole. This difference in short-axis area between the atrial and ventricular sides of the AV plane is the anatomical basis for the generation of a hydraulic force in the apex-to-base direction during diastole. The hydraulic force aids LV lengthening during diastole and facilitates AV plane displacement toward its end-diastolic position. The effect of the hydraulic force works in parallel with the restoring forces generated by ventricular contraction, which are released during diastole. The main difference between the two mechanisms is that restoring forces are mainly generated on a molecular level within the myocardium, whereas hydraulic forces are generated on the macroscopic level and are a consequence of the diastolic blood chamber pressure acting upon the anatomic surfaces of the heart. The estimation and comparison of the hydraulic force with the previously estimated peak driving force show that they are of the same order of magnitude. The effect of the hydraulic force is illustrated in a physical model (Fig. 1 and Supplemental Video S1), and the hydraulic force magnitude was found to be an important component of the total force responsible for left ventricular filling. These findings bring new insights into the physiological mechanisms governing diastolic function.

The size of the LA and the LV vary during the cardiac cycle. Since the hydraulic force is proportional to the time-varying difference between ASA and VSA (assuming negligible pressure differences between the LA and the LV), changes in ASA and VSA during LV filling influence the magnitude of the hydraulic force. If considering VSA_Epi_, hydraulic forces act during the entire duration of diastole. If considering VSA_Endo_, they act during 75% of the duration of diastole, starting immediately after the rapid filling phase. The hydraulic force is highest at the end of diastole when ASA and VSA differ the most. The elastic recoil and the hydraulic force complement each other since the hydraulic force is maximal at the end of diastole when the elastic recoil has diminished. Stored elastic energy and hydraulic forces both contribute to LV diastolic filling. The theoretical background previously used to measure and calculate recoil forces is based on measurement of the inflow to the LV across the mitral valve^20^. Notably, this methodology cannot differentiate between the respective contributions of elastic recoil and hydraulic forces. That said, Pascal’s principle provides a straightforward method for computing hydraulic forces as the product of pressure and area. Importantly, our data clearly show that the peak driving forces otherwise attributed to recoil, and hydraulic forces, respectively, are of a similar order of magnitude.

As long as there is a difference in short-axis area between the LA and the LV, there will be a force pushing the AV plane towards the base of the heart despite equal pressure in the LA and LV. This requires that in order to achieve a momentarily static equilibrium position of the AV plane during diastasis^8^,^26^, other forces, such as passive tension, must oppose the hydraulic forces.

Many industrial systems exploit Pascal’s principle to generate a large force through a large area by applying a smaller force to a smaller area. Examples of such devices are the hydraulic press, hydraulic lifts, and hydraulic brakes. The particular shape of the heart could be the result of an evolutionary adaptation that enabled the generation of such forces. Based on observations regarding the geometry of the heart, others have constructed and described a mechanical pump with two chambers (representing the LA and LV) and two valves (representing the mitral and aortic valve), and for which filling is based on hydraulic forces generated by a difference in area between the upper and the lower chamber^22^. Experiments with this mechanical pump showed fluid dynamics similar to the human heart^22^, supporting the concept of hydraulic forces contributing to diastolic filling. Similarly, simulation results have shown physiological pressures and flow in the LV and aorta, respectively, when considering hydraulic forces as the sole contribution to diastolic filling^25^.

Several studies have highlighted the importance of changes in the size of cardiac chambers in heart failure^27^,^28^,^29^. Both changes in the LV and LA geometry influence cardiac function. An increase in ASA (i.e. atrial enlargement) while preserving the same VSA, would result in a reduced hydraulic force and consequently a reduced LV filling and impaired diastolic function. The ASA is usually not measured in clinical practice. However, a meta-analysis of longitudinal data showed that an increase in left atrial long-axis area is a strong predictor of outcome in heart failure patients^30^. The chain of events causing an increase in atrial size is complex and multifactorial. One mechanism involves an increase in filling pressure that enlarges the atrium^29^. If this causes an increase in short-axis cross-section area as well, it would contribute to the reduction of the hydraulic force, preventing the LV from fully expanding longitudinally and therefore impairing diastolic filling. Importantly, conditions with enlarged atria and preserved ventricular size include restrictive and constrictive cardiomyopathies. These are disease states characterized by the presence of diastolic dysfunction. An increase in atrial size may certainly be a consequence of LV diastolic dysfunction. However, the current findings illustrate that an isolated increase in atrial size with an unchanged ventricular size will by definition reduce hydraulic forces and impair diastolic function independently of the underlying pathology in the ventricular myocardium or pericardium. This finding highlights the difficulty in inferring the direction of causality between increased left atrial size and LV diastolic dysfunction. Consequently, it may be of value to re-examine the use of increased left atrial volume index as a diagnostic criteria in the clinical assessment of diastolic dysfunction^31^.

Our study underscores the importance of maintaining a normal difference in short-axis area between the LA and the LV in order to generate adequately strong hydraulic forces to contribute to normal diastolic filling. Notably, the balance between ASA and VSA contributes to the forces involved in LV filling, but also the forces involved in LA contraction. Further studies on the consequences of pathological disruption or surgical intervention on this balance are justified^32^.

The contributions of the aortic root and aortic valve to the hydraulic forces of LV diastolic filling deserve to be addressed. Firstly, the aortic root is a structure that is integrated into the basal septal part of the LV. As such, the anatomical presence of the root entails that it is a part of the short-axis area of the LV, the VSA. The blood pressure during diastole inside the aortic root *per se* is not relevant to the LV hydraulic forces as long as the aortic valve is closed, thus separating the effects of the different pressures in the LV and aortic root. The hydraulic forces acting on the surface of the closed aortic valve from the lumen of the LV are independent of the pressure inside the aorta. The aortic root can be viewed as a pressurized bottle closed with a cap, where the cap is the aortic valve. If one tries to move the bottle, the pressure in the bottle does not make the bottle more or less difficult to move, as long as the bottle is closed. The aortic root follows the movement of AV plane towards the base, but the hydraulic force in the LV does not depend on the pressure in the aortic root, as long as the aortic valve remains closed. The relationship between the atrium and the ventricle with regards to hydraulic forces differs from the relationship between the ventricle and the aorta. Since the mitral valve is open during diastole, the atrium and ventricle are, hydraulically speaking, a single chamber. The blood pressure acts everywhere in the two contiguous chambers enabling the movement of the AV plane. Furthermore, the aorta is not unattached to other structures, and thus the aortic structure as such, including the stretch of the walls of the aorta and the great vessels, has been speculated to play a role in diastolic filling^33^. During systole, the aorta including the aortic root is pulled in the direction of the LV apex. By comparison, any stretching of the aorta during systole will result in a recoil contributing to the return of the AV-plane away from the apex during diastole. The role of these aortic-wall-related recoil forces with regards to diastole are difficult to quantify, and are beyond the scope of the current study. Taken together, importantly, the relatively high blood pressure in the aorta during diastole does not directly impact the hydraulic forces inside the LV during diastole.

The number of subjects participating in this study (n = 10) was limited. However, the magnitude of mean differences in VSA and ASA, and level of statistical significance (Fig. 3) show that the study was amply powered for quantitatively determining the differences described.

The LA has an irregular shape and therefore it can be challenging to identify and measure the largest atrial short-axis in cardiovascular magnetic resonance (CMR) images. Additionally, the atrial wall is thinner than the ventricular wall (approximately 2 mm vs 10 mm) and its thickness is typically not conspicuously visualized in CMR images due to limitations in spatial resolution. However, given its limited thickness, the atrial wall has a negligible impact on the accuracy of measuring the ASA and subsequently estimating the hydraulic forces. In order to minimize the error in the ASA measurement, we included the atrial appendage when visible and delineated the atrial contour in multiple slices. The single largest area from the atrial slices was then selected as the ASA to be compared with VSA.

When computing the hydraulic force, we do not take into account the transmitral and intraventricular pressure gradient present during early rapid filling^34^,^35^, and we assume the same pressure in the left atrium and within the left ventricular cavity, since there is no atrioventricular or intraventricular pressure gradient present during diastasis. This assumption is based on the consideration that the major contribution of hydraulic forces occurs during diastasis, when the difference in area is the largest and the pressure gradients within the LV no longer are present.

We have measured the VSA both as the endocardial and epicardial border of the LV myocardium. The rationale for using the endocardial border is that the blood is in direct contact with this myocardial border. The rationale for using the epicardial border is that both the interstitial pressure within the myocardium^36^ and the blood pressure in the coronary arteries, which is highest during diastole, may generate intramyocardial erectile hydraulic forces contributing to filling^23^,^24^. Furthermore, by including the myocardium in VSA_epi_, we have assumed that the LV intraluminal blood pressure acts upon the entire cross-section of the LV and neglected the complex myocardial structure. This assumption is difficult to validate with clinical or experimental methods since the myocardium consists of both fluids (blood, interstitial fluid and lymph) and solids (tissue fibers). Measurements of fluid pressure require invasive techniques. The estimation of the fiber stress distribution within the myocardium is also challenging, and this complexity is related to a number of factors. First, when active relaxation takes place the myocardium continuously changes its mechanical properties. Second, the myocardium is anisotropic and inhomogeneous^37^,^38^. Third, the myocardium is in motion, which can generate a complex dynamic mechanical response. Furthermore, precise quantification of forces in the longitudinal direction requires estimation of the apex-to-base component, but not the radial or circumferential component, of myocardial stress. Efforts are being made by others to model cardiac mechanics in order to provide information about stress within the myocardium^39^. Notably, the combined contribution of fluid pressure and myocardial fiber stress cannot currently be reliably estimated to be either higher or lower than the intraluminal blood pressure, and both alternatives are theoretically possible considering the current limitations in knowledge. Taken together, hydraulic forces act with certainty at least on the VSA_Endo_, and it is likely that the addition of the intramyocardial hydraulic forces render the total effective VSA to be somewhere between VSA_Endo_ and VSA_Epi_.

Furthermore, VSA_Epi_ is reduced during systole (Fig. 2). This may be counterintuitive in light of the relative constancy of the total heart volume over time. However, the total heart volume has been shown to reduce on average 8% over the cardiac cycle, and this was shown to mostly be due to epicardial changes at the ventricular level^40^. The epicardial displacement is not symmetric, but is in the form of a ‘crescent’ when end systolic and end diastolic epicardial contours are compared^41^, and represents the lateral contribution to LV stroke volume^41^. The LV epicardial volume change over the cardiac cycle has been shown to be on average 25% by volume^42^. Moreover, the healthy LV has been shown to reduce the septal portion of the LV epicardial dimensions during the cardiac cycle, contributing to approximately 5% of LV stroke volume^43^. Taken together, the change in epicardial VSA during the cardiac cycle found in the current study is of a relatively small magnitude, but consistent with previous findings.

In conclusion, a difference in short-axis area between the LV and LA proves that a hydraulic force acts on the AV plane during LV diastolic filling even in the absence of an AV pressure gradient. This force is a consequence of cardiac anatomy and assists the apex-to-base movement of the AV plane during diastolic filling of the LV. Comparison of the hydraulic force with other diastolic mechanisms, such as restoring forces, indicates that they are of comparable magnitude. This new physiological insight underscores the importance of both atrial and ventricular chamber size in the clinical assessment of diastolic function and subsequent determination of prognosis.

## Methods

### Theoretical Background Related to Hydraulic Forces

A hydraulic force is generated by the blood pressure acting upon the walls of the atrium and ventricle, and is determined by the heart’s macroscopic geometry. In the basal region of the heart, the atria are fixed in the mediastinum by the incoming vessels (caval and pulmonary veins), while the apical region is not fixed, but has been shown to exhibit negligible longitudinal movement during the cardiac cycle^44^. The AV plane can be defined as the region of transition between the ventricles and the atria. It is composed of the collagen-rich fibrous skeleton of the heart (i.e. the rings surrounding the four cardiac valves) as well as the muscles attached to it. The attachment of the muscles to the skeleton of the heart creates a dome-shaped surface that can be compared to the head of a piston. This complex semi-rigid AV plane easily slides along the pericardium in the longitudinal (apex-base) direction, since the pericardium is lubricated by the pericardial fluid, which allows for near frictionless contact and motion between the two structures. This back-and-forth motion of the AV plane in the apex-base direction is similar to that of a piston that displaces blood during its motion^22^,^45^, as clearly visible in CMR cine images^44^. The piston-like movements of the AV plane allow for reciprocal volume changes between the atria and the ventricles while keeping an approximately constant total heart volume^40^,^46^. During diastole, the mitral valve is open and pressures in the LV cavity and in the left atrium (LA) are effectively the same. This pressure acts in all directions against the myocardium and valve structures, from the atrial side of the AV plane, and from the ventricular side of the AV plane.

**Figure 4:**
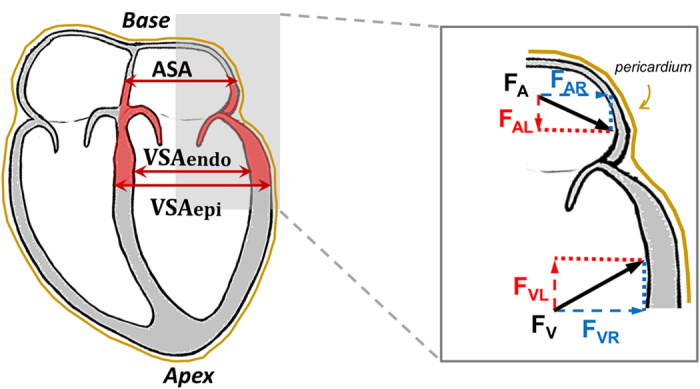
Hydraulic forces acting on the myocardium. On the left, schematic four-chamber view of the heart where the AV plane region of the left heart is highlighted in red. The left-atrial short-axis area (ASA), the left-ventricular short-axis area at the endocardial border (VSA_Endo_) and the left-ventricular short-axis area at the epicardial border (VSA_Epi_) are indicated. During diastole the mitral valve is open and the pressure in the left atrium (LA) and left ventricle (LV) is effectively the same. This pressure generates a force acting perpendicularly to the myocardial wall at each location. On the right side of the figure, the force in the atrium corresponds to F_A_ and the force is the ventricle corresponds to F_V_. These two forces can be resolved into their components in the longitudinal base-apex direction (F_AL_ and F_VL_) and in the radial direction (F_AR_ and F_VR_). The radial components of the hydraulic force, F_AR_ and F_VR_, are partially counteracted by the presence of the pericardium and the surrounding tissues, and may contribute to radial and torsional movement of the myocardium during diastole. The longitudinal components (F_AL_ and F_VL_) are the hydraulic forces presented in this study, and they act on the longitudinally oriented projection of F_A_ and F_V_, as indicated with dashed red lines, which correspond to the largest cross-section of the LV and LA, respectively. This illustrates the reason for choosing the largest short-axis area in the LA and in the LV to estimate the hydraulic force in the apex-base direction in the heart.

Visual inspection of CMR images gives the impression that ASA is smaller than VSA. Short-axis area is defined as the cross-section obtained by cutting the LA or LV with a plane parallel to the AV plane (Fig. 4). Since force is pressure times area, the same pressure applied over different areas generates different forces. Liquids are nearly incompressible, therefore the pressure is transmitted without losses and generates different magnitudes of force depending on differences in surface area. This is referred to as Pascal’s principle. Forces transmitted through a liquid are referred to as hydraulic forces.

The effect of hydraulic force on a curved surface is composed of two orthogonally directed force components (vectors), namely, the longitudinal component acting in the base-apex direction, and the radial component acting orthogonally to the longitudinal component (Fig. 4). For the purposes of understanding the effects of hydraulic forces upon LV diastolic filling, only the longitudinal component of the hydraulic force is relevant (F_AL_ and F_VL_ in Fig. 4). The magnitude of this longitudinal component is determined by the cross-sectional area orthogonal to the longitudinal direction. By comparison, in order to calculate the longitudinal component of the hydraulic force acting on the curved walls of a submerged container, it is only necessary to know the widest area of the container, and this is independent of the curvature of the surface.

The exact anatomy of the AV plane is complex and includes the mitral valve orifice which is often described as having the shape of a saddle. Notably, the exact three-dimensional geometry of the AV plane is less relevant when it comes to understanding the hydraulic forces acting upon the AV plane in the apex-base direction. From the perspective of the physics of these apex-to-base directed forces, the geometry of relevance is reduced to the largest short-axis areas, orthogonal to the apex-base direction, which exist on either sides of the AV plane. The resultant force component along the apex-to-base direction can by definition only act on the effective area orthogonal to the direction of the force, regardless of the three-dimensional geometry of the anatomy. The AV plane’s largest area on the ventricular side corresponds to the VSA and the largest area on the atrial side to the ASA.

**Figure 5:**
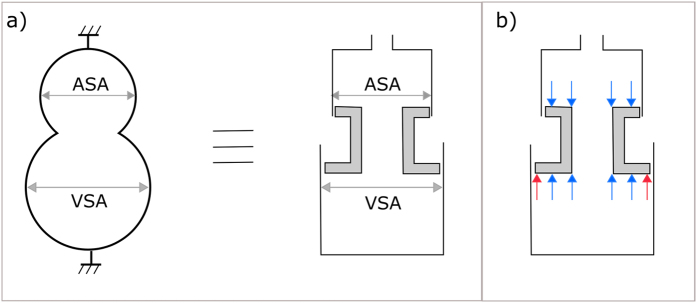
Schematic illustration of the left side of the heart. (**a**) Note that the atrial short-axis area (ASA) is smaller than the ventricular short-axis area (VSA). This representation of the left heart is equivalent to a simplified construction where the two chambers have a constant cross-section and a movable hollow piston placed in between. (**b**) Forces acting on the two sides of the piston. The arrows represent the forces exerted upon by the blood pressure on the surface of the heart. The blue arrows counterbalance each other, whereas the read arrows represent the net force which pushes the piston upwards. The magnitude of the force pushing the piston upwards is proportional to the difference in area (VSA minus ASA).

Based on the aforementioned considerations, if we consider the AV plane as a piston with a central lumen and two different areas (Fig. 5a and b), one representing the ASA and one representing the VSA, we can calculate the hydraulic force (F) acting on this piston, in the apex-to-base direction, according to the formula F = (VSA × Ventricular Pressure) − (ASA × Atrial Pressure). Thus, a hydraulic force can contribute to the diastolic apex-to-base movement of the AV plane if ventricular and atrial short-axis areas are different, even if the ventricular and atrial pressures are equal.

It should be noted that pressure is a non-directional magnitude, a scalar, whereas force is a vector acting in a given direction. When referring to hydraulic force in this article, we are referring to the apex-to-base directed component of the resultant force generated by the pressure in the LV and LA on the myocardium.

### Physical Model of the Hydraulic Forces aiding Left Ventricular Filling

In order to illustrate the effect of hydraulic forces displacing a movable piston with different areas, we built a physical model of the LA and LV, which is shown in Fig. 1. A video showing the movement of the piston under the action of hydraulic forces is available as an online supplemental file accompanying this article.

### *In Vivo* Measurements

Data were obtained from 10 healthy volunteers (5 women, age 24 ± 3 years) which have previously been studied for other purposes^44^. The study was approved by the Regional Ethics Committee in Lund (Regionala Etikprövningsnämnden i Lund), Sweden, with approval number 269/2005 and performed in accordance with relevant guidelines and regulations. All subjects provided written informed consent. The volunteers underwent CMR with steady-state free precession cine images using a 1.5 T scanner (Philips Intera, Philips, Best, Netherlands) with 30 frames per heart cycle. The details of the CMR image acquisition parameters have been previously described^44^. The angulation and positioning of the short-axis imaging plane was performed according to standardized techniques whereby the short-axis imaging plane was positioned orthogonal to the long-axis imaging plane as determined in two mutually orthogonal long-axis images^47^.

### Atrial Short-Axis Area (ASA) and Ventricular Short-Axis Area (VSA) Measurements

**Figure 6:**
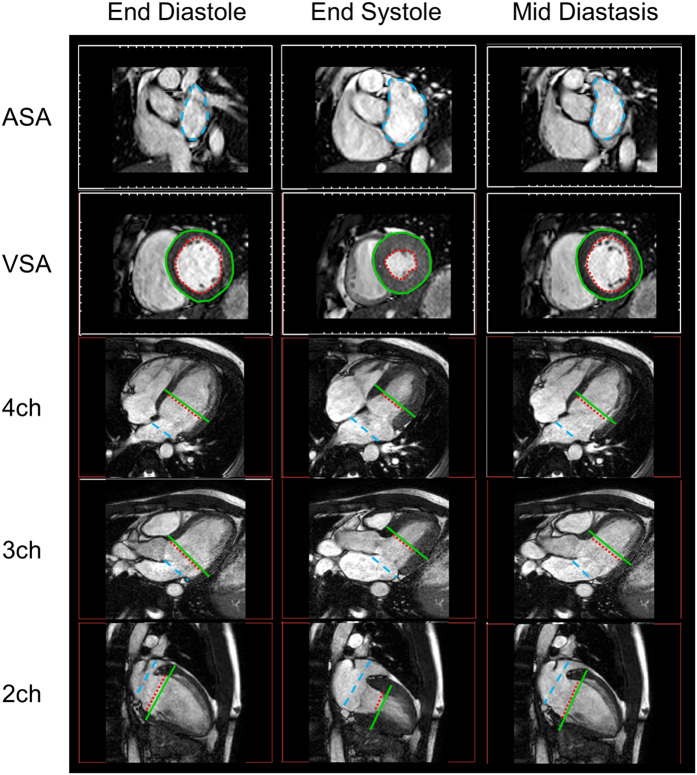
Magnetic resonance (MR) images from a representative subject. From top to bottom, short-axis view at the level of the left atrium, short-axis view at the level of the left ventricle, four-chamber view (4 ch), three-chamber view (3 ch), and two-chamber view (2 ch). The five views are shown at end diastole, end systole and the mid diastasis phase of the cardiac cycle. The dashed blue line is the manually delineated atrial short-axis area (ASA), the dashed red line is the endocardial ventricular short-axis area (VSA_Endo_) and the continuous green line is the epicardial ventricular short-axis area (VSA_Epi_). The short-axis view corresponds to the imaging plane at the level of the blue, respectively green and red, lines in the four-chamber view, three-chamber view and two-chamber view.

CMR images were analyzed using the freely available software Segment (version 2.0 R4636, Medviso AB, Lund, Sweden)^48^. For each subject, the ASA, including the atrial appendage when visible, was manually segmented in multiple short-axis slices at each time point in the cardiac cycle (Fig. 6). The VSA was at first automatically segmented using the Segment software function for automatic segmentation of the LV, and the contours were subsequently manually adjusted for accuracy. The VSA was segmented both excluding (VSA_Endo_) and including (VSA_Epi_) the LV myocardium. The VSA segmentation was performed in multiple short-axis slices in the vicinity of the AV plane and the largest endocardial and epicardial area at each time point was selected as VSA_Endo_ and VSA_Epi_, respectively.

### Comparison between Peak Driving Force and Hydraulic Force

To understand the relative contribution of hydraulic forces in relation to the peak driving force of LV filling, we sought to estimate the magnitude of the peak driving force and the hydraulic force. The peak driving force per unit mass during diastolic filling has been previously estimated in a population of young healthy subjects and is equal to approximately 25 mN/g^49^. Notably, this force is calculated per unit inertial load in grams, i.e. per unit of mass that needs to be moved by the force.

In mechanics and physiology, inertial load is mass accelerated by a force. In the heart, the diastolic inertial load is the total mass of myocardium and blood set in motion as a result of myocardial relaxation that unmasks the stored elastic strain powering left ventricular recoil during diastole. Quantification of inertial load is challenging. Conceptually, inertial load includes the left ventricular myocardium as well as the blood accelerated from a resting position during early rapid filling, but also includes motion of the roots of the great vessels with closed valves.

In order to estimate an approximate range of the inertial load, we used the LV mass of young healthy subjects, which on average has been shown to be approximately 125 g^50^. To include the additional contribution of the blood and the roots of the great vessels, we estimated it to range between a minimum of 75 g and a maximum of 275 g, yielding a total inertial load ranging between 200 and 400 g. Consequently, an estimate of the range of the peak driving force of LV filling was calculated as 25 mN/g ∙ 200–400 g inertial load, yielding a range of 5–10 N.

A hydraulic force is calculated as Force = (Pressure) × (Difference in Area). Mean LV diastolic pressure was estimated from normal individuals to be 10 mmHg or equivalently 1333 Pa^51^. The hydraulic force for each subject was calculated by multiplying by the LV mean diastolic pressure with the difference (VSA_Endo_ − ASA) and (VSA_Epi_ − ASA) at mid diastasis, respectively.

### Statistical Analysis

Values obtained for VSA_Endo_ and VSA_Epi_ for each individual at a specific time point were compared with the corresponding ASA. All data are expressed as mean ± SEM. The difference between VSA and ASA was tested using the Wilcoxon signed rank test. The statistical analysis was performed using MATLAB (version R2014a, Mathworks, Inc., Natick, MA, USA) and a difference with a p-value < 0.05 was considered statistically significant.

## Additional Information

**How to cite this article**: Maksuti, E. *et al*. Hydraulic forces contribute to left ventricular diastolic filling. *Sci. Rep.* **7**, 43505; doi: 10.1038/srep43505 (2017).

**Publisher's note:** Springer Nature remains neutral with regard to jurisdictional claims in published maps and institutional affiliations.

## Supplementary Material

Supplementary Video S1

Supplementary Information
