# Supplementary material for: Hydraulic forces contribute to left ventricular diastolic filling
Source: Sci Rep. 2017 Mar 3;7:43505. doi: 10.1038/srep43505 (PMC5334655; doi:10.1038/srep43505)
Supplement: Supplementary Information [file srep43505-s2.pdf]

Hydraulic forces contribute to left ventricular diastolic filling

Elira Maksuti[1,2], Marcus Carlsson[3], Håkan Arheden[3], Sándor J. Kovács[4], Michael Broomé[2,5,6], Martin Ugander[1]

[1]Department of Clinical Physiology, Karolinska Institutet, and Karolinska University Hospital, Stockholm, Sweden.

[2]Department of Medical Engineering, School of Technology and Health, KTH Royal Institute of Technology, Stockholm, Sweden.

[3]Lund University, Skane University Hospital, Department of Clinical Sciences Lund, Clinical Physiology, Lund, Sweden.

[4]Department of Internal Medicine, Cardiovascular Division, Washington University School of Medicine, St. Louis, Missouri, USA.

[5]Anaesthesiology and Intensive Care, Department of Physiology and Pharmacology, Karolinska Institutet, Stockholm, Sweden.

[6]ECMO Department, Karolinska University Hospital, Stockholm, Sweden.

Supplementary Materials:

Video S1

Description: The physical model with a hollow moving piston illustrating hydraulic forces analogous to those present in the left atrium and left ventricle of the heart. See figure text for Figure 1 in the main article.
